# Relationship between blood urea nitrogen and 28-day all-cause mortality in patients with acute pulmonary edema: a retrospective analysis of the MIMIC-IV database

**DOI:** 10.3389/fcvm.2025.1569218

**Published:** 2025-08-05

**Authors:** Guang Tu, Yuwen Liu, Xiaomi Huang, Yanfang Zeng, Zhonglan Cai, Chunyan Wang

**Affiliations:** ^1^ Department of Cardiology, Lichuan People’s Hospital, Fuzhou, China; ^2^ Department of Cardiovascular Medicine, Suizhou Hospital, Hubei Medicine University, Suizhou, China; ^3^ Department of Cardiology, Qixingguan District People’s Hospital, Bijie, China; ^4^ Department of Cardiovascular Medicine, Shanghai Public Health Clinical Center (Fudan University), Shanghai, China

**Keywords:** acute pulmonary edema, blood urea nitrogen, 28-day all-cause mortality, prognosis, MIMIC-IV database

## Abstract

**Background:**

Acute pulmonary edema is a severe clinical syndrome with high mortality. Blood Urea Nitrogen (BUN) levels, which indicate renal function and metabolic state, may have prognostic value in critically ill patients. However, their relationship with outcomes in acute pulmonary edema remains unclear.

**Objective:**

This study aims to investigate the association between admission BUN levels and 28-day all-cause mortality in patients with acute pulmonary edema.

**Methods:**

This retrospective cohort study utilized data from the Medical Information Mart for Intensive Care IV (MIMIC-IV) database, covering the period from 2008–2019. It included adult patients diagnosed with acute pulmonary edema. Patients were divided into four groups based on their BUN levels. Cox regression models, restricted cubic spline (RCS) curves, Kaplan–Meier analysis, and subgroup analyses were used to assess the relationship between BUN levels and mortality.

**Results:**

A total of 1,094 patients were included in the study. Univariate Cox regression analysis revealed a positive correlation between BUN levels and 28-day mortality (HR = 1.02, 95% CI: 1.01–1.02, *P* < 0.001). Multivariate analysis confirmed BUN as an independent predictor of mortality (HR = 1.02, 95% CI: 1.01–1.02, *P* < 0.001). The RCS curve indicated a nonlinear relationship, and Kaplan–Meier analysis showed lower survival in the higher BUN groups (*P* < 0.001). Subgroup analysis found the association to be significant across all subgroups.

**Conclusion:**

Admission BUN levels predict 28-day all-cause mortality in patients with acute pulmonary edema. Clinically, BUN monitoring should be emphasized, and individualized prognostic and treatment strategies should be developed to improve outcomes.

## 1 Introduction

Acute pulmonary edema is a critical clinical condition characterized by the rapid onset of respiratory distress due to the accumulation of fluid in the alveoli (1). It is commonly associated with severe heart failure, particularly left ventricular dysfunction, and can lead to significant morbidity and mortality if not promptly managed (2). The pathophysiology of acute pulmonary edema involves increased pulmonary capillary pressure, leading to the transudation of fluid into the lung interstitium and alveoli (3). Early identification and intervention are crucial to improve outcomes, yet the clinical presentation can be variable and challenging to assess accurately.

Blood Urea Nitrogen (BUN) levels have emerged as an important biomarker in the evaluation of critically ill patients (4). BUN is produced when nitrogenous waste products are not efficiently cleared by the kidneys, leading to its accumulation in the blood (5). Elevated admission BUN levels are indicative of renal dysfunction and have been shown to correlate with adverse outcomes in various clinical settings, including sepsis, trauma, and cardiac arrest (6–8). However, the role of admission BUN as a prognostic marker in patients with acute pulmonary edema remains less explored. Previous studies have primarily focused on BUN levels in the context of renal failure and sepsis, where its predictive value for mortality has been well established (9, 10). In patients with acute pulmonary edema, the relationship between BUN levels and clinical outcomes, particularly mortality, is not well defined. Understanding this relationship could provide valuable insights into the severity of the condition and help guide therapeutic decisions.

The MIMIC-IV database offers a unique opportunity to investigate this association due to its comprehensive and detailed clinical data from a large cohort of patients (11, 12). By analyzing the data from this database, we aim to determine whether BUN levels at admission can predict 28-day all-cause mortality in patients with acute pulmonary edema. This study seeks to fill a gap in the literature and potentially enhance the prognostic assessment of patients presenting with this condition.

## 2 Methods

### 2.1 Study design

This study is a retrospective cohort analysis utilizing data from the MIMIC-IV database. The primary objective was to investigate the association between BUN levels at admission and 28-day all-cause mortality in patients with acute pulmonary edema. The MIMIC-IV database is a publicly available, large-scale, multicenter electronic health record database that includes detailed clinical data, providing a rich resource for this analysis.

### 2.2 Data source

The data were sourced from the MIMIC-IV database, which is collected and maintained by Massachusetts General Hospital and covers patient data from 2008–2019 (12). Author Guang Tu finished the CITI Data or Specimens Only Research course, obtained approval for database access, and assumed responsibility for data extraction (certification number 65828445). As the study utilized an anonymous public database in compliance with the review board's protocol, ethical consent was deemed unnecessary.

### 2.3 Study population

The study population consisted of adult patients diagnosed with acute pulmonary edema in the MIMIC-IV database. Inclusion criteria included patients aged 18 years or older with a clinical diagnosis of acute pulmonary edema at admission and BUN level measurements within the first 24 h of hospitalization. Exclusion criteria were patients with chronic pulmonary edema, cardiogenic shock, severe trauma, or major surgery prior to admission, as well as those with incomplete data.

### 2.4 Data collection

**Figure 1:**
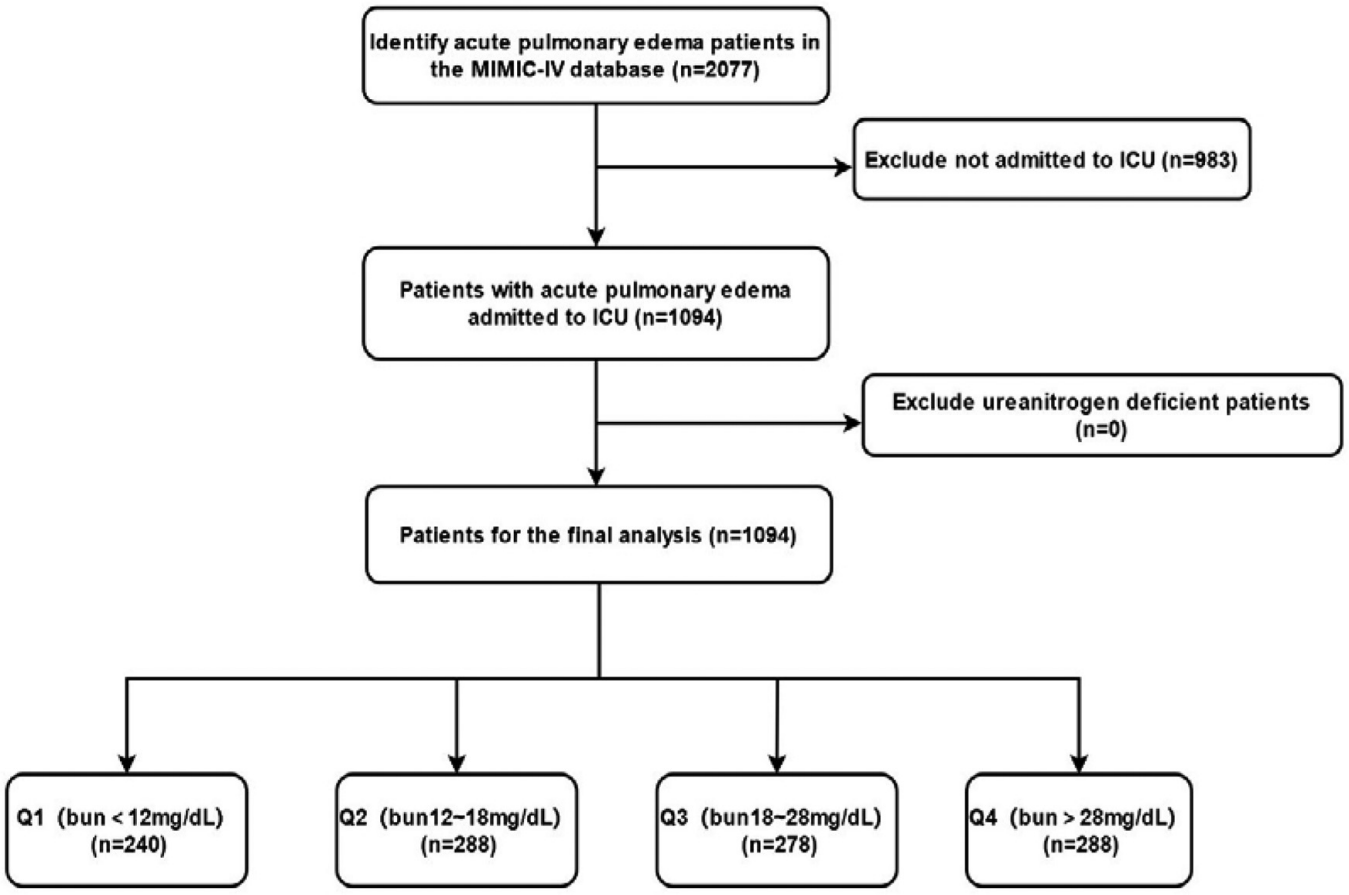
Flowchart of patient inclusion.

Data were extracted from the MIMIC-IV database, including baseline demographic and clinical characteristics of the patients. These included gender, age, race, past medical history (such as myocardial infarction, heart failure, cerebrovascular disease, chronic lung disease, diabetes, renal disease, etc.), comorbidities, vital signs [systolic blood pressure (SBP), diastolic blood pressure (DBP), heart rate, etc.], laboratory test results [white blood cell count (WBC), red blood cell count (RBC), platelet count, hemoglobin levels, blood glucose, calcium, potassium, sodium levels, etc.], and admission BUN levels. All data were stored in electronic health records, and the research team used professional data extraction tools and procedures to obtain and organize the data (Figure 1).

### 2.5 Grouping method

Patients were divided into four groups (Q1–Q4) based on their BUN levels, with each group containing approximately 25% of the patients. The specific grouping criteria were as follows: Q1 group (BUN < 12 mg/dl), Q2 group (BUN 12–18 mg/dl), Q3 group (BUN 18–28 mg/dl), and Q4 group (BUN > 28 mg/dl). This grouping method facilitated the observation of the different impacts of BUN levels on patient outcomes and provided convenience for subsequent statistical analysis.

### 2.6 Statistical analysis

All statistical analyses were conducted using R Statistical Software (Version 4.2.2). A two-sided *p*-value threshold of less than 0.05 was set to determine statistical significance.

Initially, descriptive statistical analysis was performed on the patients' baseline characteristics, with continuous variables expressed as mean ± standard deviation (SD) or median (interquartile range) and categorical variables as frequency and percentage. Group comparisons were made using independent samples *t*-test or Mann–Whitney *U* test (for continuous variables) and chi-square test or Fisher's exact test (for categorical variables) to assess differences in baseline characteristics among the different BUN level groups.

Subsequently, univariate Cox regression models were employed to analyze the relationship between BUN levels and 28-day all-cause mortality in patients with acute pulmonary edema, calculating hazard ratios (HR) and their 95% confidence intervals (CI). Multivariate Cox regression models were then constructed, adjusting for confounders such as age, gender, race, comorbidities, and variables significant at *P* < 0.050 in the univariate Cox regression analysis, to further validate the status of BUN as an independent risk factor for 28-day all-cause mortality.

Additionally, restricted cubic spline (RCS) curves were utilized to evaluate whether there was a nonlinear relationship between BUN levels and 28-day all-cause mortality, providing a more intuitive display of the impact trend of BUN levels on mortality risk. Kaplan–Meier survival analysis curves were employed to compare the survival rate differences among different BUN level groups, with Log-rank tests used for statistical comparisons.

Finally, subgroup analyses were conducted to explore the stability of the relationship between BUN levels and 28-day all-cause mortality in different subgroups (such as age, race, gender, comorbidities, etc.). Forest plots were created to display the HR and 95% CI for each subgroup, and interaction tests were performed to assess differences in the impact of BUN levels on mortality risk among different subgroups.

## 3 Results

### 3.1 Baseline demographic and clinical characteristics

A total of 1,094 patients with acute pulmonary edema were included in this study. The patients were divided into four groups (Q1–Q4) based on their BUN levels. There were significant differences in gender distribution among the groups (*P* < 0.001), with females comprising 51.4% of the total cohort. The majority of patients were white (63.5%), and racial composition did not significantly differ across the groups (*P* = 0.075). The mean age of the patients was 65.5 years, with age differences approaching statistical significance among the groups (*P* < 0.001). Systolic blood pressure (SBP) varied significantly across the groups (*P* = 0.013), while diastolic blood pressure (DBP) did not (*P* = 0.114). Heart rate also showed significant differences among the groups (*P* < 0.001). The prevalence of past medical history, including myocardial infarction, heart failure, cerebrovascular disease, chronic lung disease, diabetes, and renal disease, did not significantly differ among the groups (*P* > 0.050). However, there were significant differences in white blood cell count (WBC), red blood cell count (RBC), platelet count, and hemoglobin levels among the groups (*P* < 0.050), while blood glucose, calcium, potassium, and sodium levels did not (*P* > 0.050) (Table 1).

**Table 1:** General characteristics of patients with acute pulmonary edema.

Variables	Total	Q1	Q2	Q3	Q4	*P* _value
	(*n* = 1,094)	(*n* = 240)	(*n* = 288)	(*n* = 278)	(*n* = 288)	
Gender, *n* (%)						<0.001
Female	562 (51.4)	149 (62.1)	154 (53.5)	123 (44.2)	136 (47.2)	
Male	532 (48.6)	91 (37.9)	134 (46.5)	155 (55.8)	152 (52.8)	
Race, *n* (%)						0.075
White	695 (63.5)	141 (58.8)	183 (63.5)	192 (69.1)	179 (62.2)	
Black	97 (8.9)	29 (12.1)	26 (9)	13 (4.7)	29 (10.1)	
Orther	302 (27.6)	70 (29.2)	79 (27.4)	73 (26.3)	80 (27.8)	
Age (year), mean (SD)	65.5 ± 16.4	58.9 ± 16.8	65.6 ± 15.8	69.8 ± 14.8	66.8 ± 16.6	<0.001
sbp (mmHg), mean (SD)	118.2 ± 17.0	115.3 ± 16.0	118.0 ± 15.5	118.8 ± 16.6	120.1 ± 19.1	0.013
dbp (mmHg), mean (SD)	63.2 ± 11.3	64.7 ± 10.2	63.1 ± 10.9	62.6 ± 11.2	62.6 ± 12.6	0.114
Heart rate(beats/min), mean (SD)	87.7 ± 16.3	92.0 ± 16.3	86.0 ± 16.0	85.6 ± 15.9	87.8 ± 16.6	<0.001
Myocardial infarct, *n* (%)						0.015
No	929 (84.9)	216 (90.0)	246 (85.4)	222 (79.9)	245 (85.1)	
Yes	165 (15.1)	24 (10.0)	42 (14.6)	56 (20.1)	43 (14.9)	
Heart failure, *n* (%)						0.011
No	984 (89.9)	221 (92.1)	270 (93.8)	244 (87.8)	249 (86.5)	
Yes	110 (10.1)	19 (7.9)	18 (6.2)	34 (12.2)	39 (13.5)	
Cerebrovascular, *n* (%)						0.070
No	944 (86.3)	207 (86.2)	244 (84.7)	232 (83.5)	261 (90.6)	
Yes	150 (13.7)	33 (13.8)	44 (15.3)	46 (16.5)	27 (9.4)	
Chronic pulmonary, *n* (%)						0.238
No	817 (74.7)	185 (77.1)	207 (71.9)	201 (72.3)	224 (77.8)	
Yes	277 (25.3)	55 (22.9)	81 (28.1)	77 (27.7)	64 (22.2)	
Diabetes, *n* (%)						0.140
No	844 (77.1)	187 (77.9)	235 (81.6)	207 (74.5)	215 (74.7)	
Yes	250 (22.9)	53 (22.1)	53 (18.4)	71 (25.5)	73 (25.3)	
Renal_disease, *n* (%)						<0.001
No	876 (80.1)	231 (96.2)	266 (92.4)	218 (78.4)	161 (55.9)	
Yes	218 (19.9)	9 (3.8)	22 (7.6)	60 (21.6)	127 (44.1)	
wbc( × 10^9^/L), Median (IQR)	9.8 (6.8, 13.4)	9.4 (6.4, 13.0)	10.1 (7.2, 13.5)	9.8 (6.8, 13.2)	9.6 (6.3, 14.6)	0.412
Red (×10^9^/L), mean (SD)	3.5 ± 0.8	3.6 ± 0.8	3.6 ± 0.8	3.6 ± 0.8	3.2 ± 0.9	<0.001
Platelets(×10^9^/L), mean (SD)	175.5 ± 101.7	187.2 ± 104.7	175.4 ± 94.4	180.7 ± 102.5	160.7 ± 104.0	0.018
Hemoglobin(mg/dl), mean (SD)	9.8 ± 2.3	9.9 ± 2.3	10.1 ± 2.4	10.1 ± 2.3	9.1 ± 2.2	<0.001
Creatinine(mg/dl), Median (IQR)	0.9 (0.7, 1.4)	0.6 (0.5, 0.8)	0.8 (0.7, 1.0)	1.0 (0.8, 1.3)	1.9 (1.4, 3.4)	<0.001
Glucose(mmol/dl), mean (SD)	120.6 ± 40.7	117.0 ± 45.4	123.4 ± 35.8	124.6 ± 40.0	116.8 ± 41.4	0.037
Calcium(mmol/dl), mean (SD)	8.1 ± 0.9	7.9 ± 1.0	8.0 ± 0.8	8.1 ± 0.8	8.2 ± 0.9	<0.001
Potassium(mmol/dl), mean (SD)	3.9 ± 0.6	3.6 ± 0.5	3.9 ± 0.5	3.9 ± 0.5	4.1 ± 0.7	<0.001
Sodium(mmol/dl), mean (SD)	136.4 ± 4.9	136.0 ± 5.1	136.5 ± 4.4	136.7 ± 4.3	136.3 ± 5.6	0.517

### 3.2 BUN as an independent risk factor for 28-day all-cause mortality

Univariate Cox regression analysis revealed a positive correlation between admission BUN levels and 28-day all-cause mortality in patients with acute pulmonary edema (HR = 1.02, 95% CI: 1.01–1.02, *P* < 0.001), indicating that for every 1 mg/dl increase in admission BUN, the risk of death increased by 2% (Table 2). Multivariate Cox regression analysis, after adjusting for confounders such as age, gender, race, comorbidities, and variables significant at *P* < 0.050 in the univariate Cox regression analysis, confirmed admission BUN as an independent risk factor for 28-day all-cause mortality (HR = 1.02, 95% CI: 1.01–1.02, *P* < 0.001). When stratified by admission BUN levels, patients in the Q3 group (admission BUN 18–28 mg/dl) and Q4 group (admission BUN > 28 mg/dl) had significantly higher mortality risks compared to the Q1 group (admission BUN < 12 mg/dl), and a dose-response relationship was observed, with higher admission BUN levels associated with greater mortality risks (*P* < 0.001) (Table 3).

**Table 2:** A univariate Cox regression model evaluated the association between blood urea nitrogen and 28-day all-cause mortality in patients with acute pulmonary edema.

Item	HR (95% CI)	*P* _value
Gender: male vs. female	0.97 (0.73, 1.28)	0.807
Race: ref. = white		
Black	0.53 (0.27, 1.05)	0.070
Orther	1.38 (1.02, 1.86)	0.038
Age (year)	1.02 (1.01, 1.03)	<0.001
sbp(mmHg)	0.97 (0.96, 0.98)	<0.001
dbp(mmHg)	0.97 (0.96, 0.99)	<0.001
Heart rate (beats/min)	1.01 (1.00, 1.02)	0.018
Myocardial infarct: yes vs. no	1.42 (1.00, 2.04)	0.053
Heart failure: yes vs. no	1.15 (0.73, 1.80)	0.553
Cerebrovascular: yes vs. no	1.07 (0.71, 1.59)	0.751
Chronic pulmonary: yes vs. no	0.75 (0.53, 1.06)	0.101
Diabetes: yes vs. no	1.03 (0.73, 1.44)	0.871
Renal disease: yes vs. no	1.24 (0.89, 1.73)	0.210
wbc(×10^9^/L)	1.01 (1.00, 1.02)	0.006
rbc(×10^12^/L)	0.83 (0.70, 0.99)	0.040
Platelets (×10^9^/L)	0.99 (0.99, 1.01)	0.085
Hemoglobin (mg/dl)	0.93 (0.87, 0.99)	0.022
bun (mg/dl)	1.02 (1.01, 1.02)	<0.001
Creatinine (mg/dl)	1.05 (0.98, 1.13)	0.194
Glucose(mmol/dl)	0.99 (0.99, 1.01)	0.972
Calcium(mmol/dl)	0.91 (0.77, 1.09)	0.312
Potassium (mmol/dl)	0.90 (0.70, 1.15)	0.395
Sodium (mmol/dl)	1.03 (1.00, 1.07)	0.037

**Table 3:** A multivariate Cox regression model evaluated the association between blood urea nitrogen and 28-day all-cause mortality in patients with acute pulmonary edema.

Variable	No	Crude model		Model 1		Model 2		Model 3	
		HR (95%CI)	*P* _value	HR (95% CI)	*P* _value	HR (95% CI)	*P* _value	HR (95% CI)	*P* _value
bun	1,094	1.02 (1.01–1.02)	<0.001	1.02 (1.01–1.02)	<0.001	1.02 (1.01–1.02)	<0.001	1.02 (1.01–1.02)	<0.001
bun (quartile)									
Q1 (<12)	240	1(Ref)		1(Ref)		1(Ref)		1(Ref)	
Q2 (12–18)	288	1.04 (0.61–1.78)	0.878	0.96 (0.56–1.64)	0.871	0.96 (0.56–1.66)	0.897	0.86 (0.45–1.65)	0.644
Q3 (18–28)	278	1.92 (1.18–3.11)	0.009	1.66 (1.01–2.73)	0.047	1.74 (1.05–2.88)	0.030	1.47 (0.81–2.65)	0.202
Q4 (>28)	288	3.33 (2.12–5.24)	<0.001	3.05 (1.92–4.83)	<0.001	3.38 (2.09–5.45)	<0.001	2.41 (1.38–4.23)	0.002
Trend.test	1,094	1.59 (1.38–1.83)	<0.001	1.57 (1.36–1.81)	<0.001	1.62 (1.39–1.89)	<0.001	1.43 (1.19–1.72)	<0.001

### 3.3 Nonlinear relationship analysis

**Figure 2:**
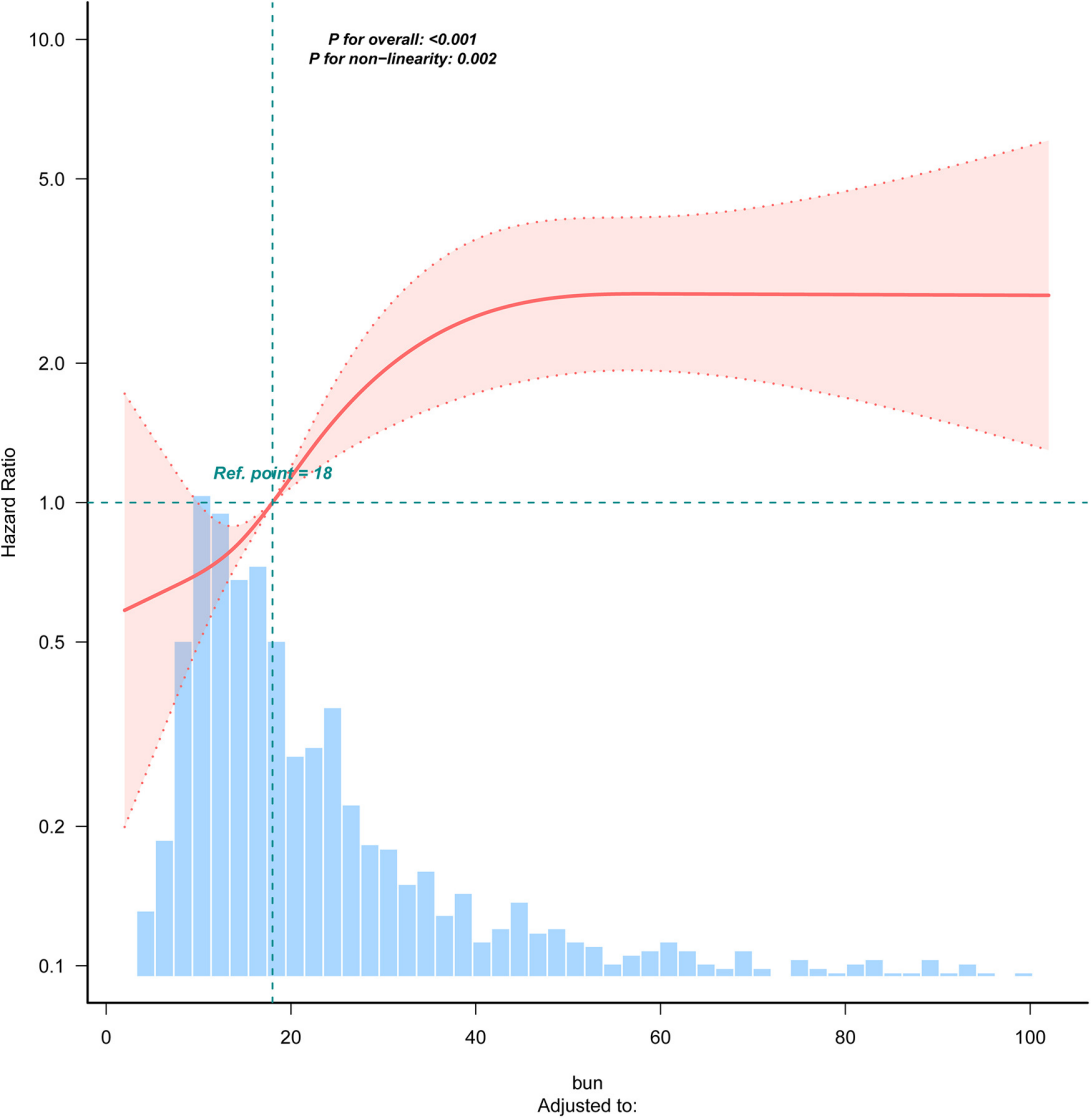
RCS curve for the blood urea nitrogen. bun (quartile): Q1 (<12), Q2 (12–18), Q3 (18–28), Q4 (>28).

Figure 2 demonstrates the nonlinear relationship between BUN levels and 28-day all-cause mortality in patients with acute pulmonary edema using a restricted cubic spline (RCS) curve. The analysis reveals that the relationship between BUN levels and mortality is not a simple linear one but rather a curve with an inflection point. Table 4 further confirms this nonlinear relationship, identifying a threshold of 38.21 mg/dl for BUN levels (95% CI: 37.528–38.893). Below this threshold, each 1 mg/dl increase in BUN is associated with a significant 4.5% increase in 28-day all-cause mortality (HR = 1.045, *P* < 0.001). However, above the threshold, further increases in BUN do not significantly affect mortality (HR = 0.9981, *P* = 0.8263). This indicates that BUN levels significantly impact mortality below the threshold but have a diminished effect above it.

**Table 4:** Cox regression model was used to analyze the threshold effect of blood urea nitrogen on 28-day all-cause mortality.

Item	HR (95% CI)	*P* value
Turning point (mg/dl)	38.21 (37.53, 38.89)	
bun <38.21	1.05 (1.02,1.07)	<0.001
bun ≧38.21	0.99 (0.98,1.02)	0.826
Likelihood Ratio test		<0.001

### 3.4 Kaplan–Meier curves

**Figure 3:**
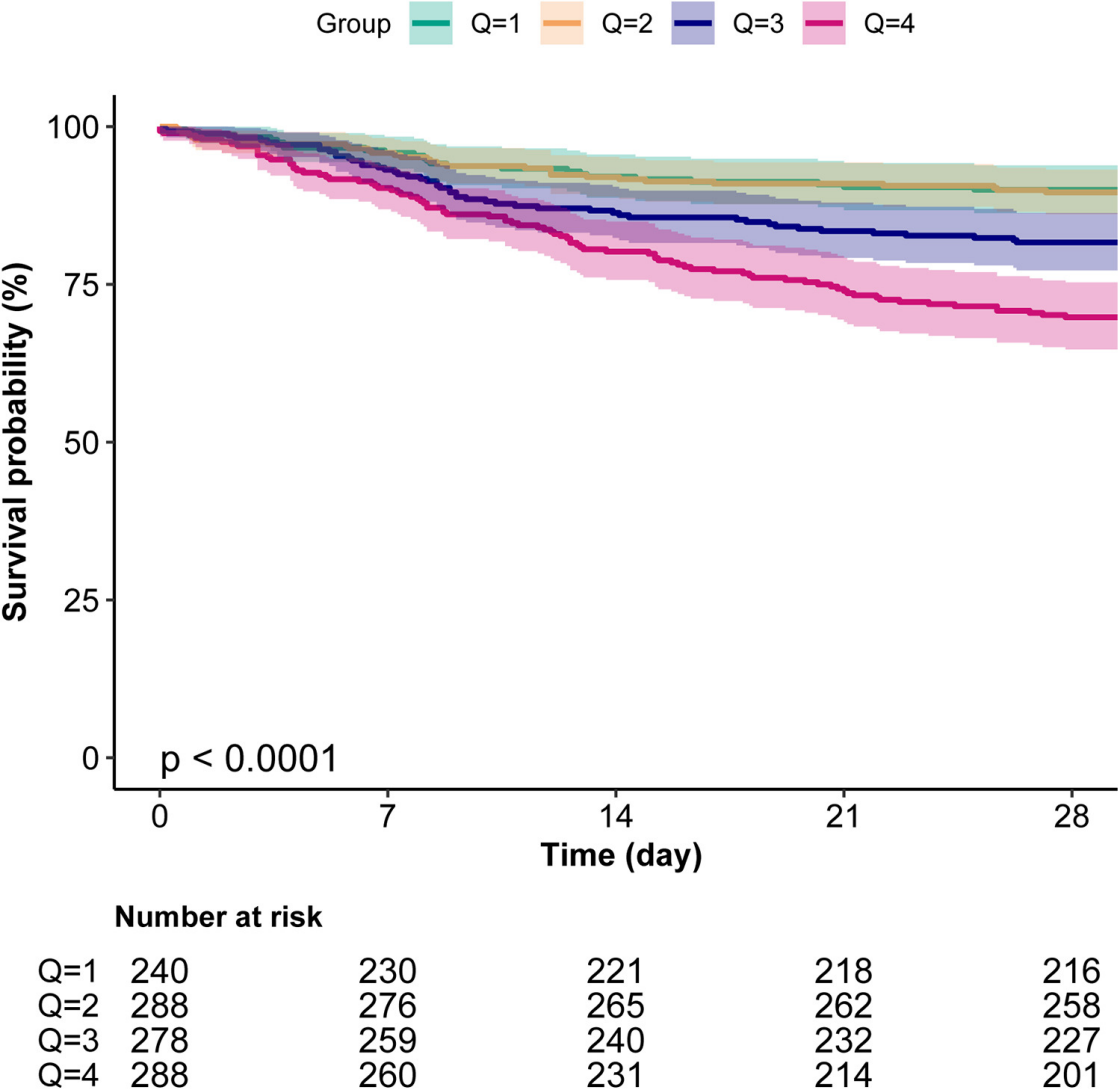
Kaplan–Meier survival analysis curves for 28-day all-cause mortality.

Kaplan–Meier survival analysis further validated the impact of BUN levels on survival prognosis, showing that the survival rate of patients in the Q4 group was significantly lower than that of other groups (*P* < 0.001), indicating a close association between high BUN levels and poor survival outcomes (Figure 3).

### 3.5 Subgroup analysis and forest plot

**Figure 4:**
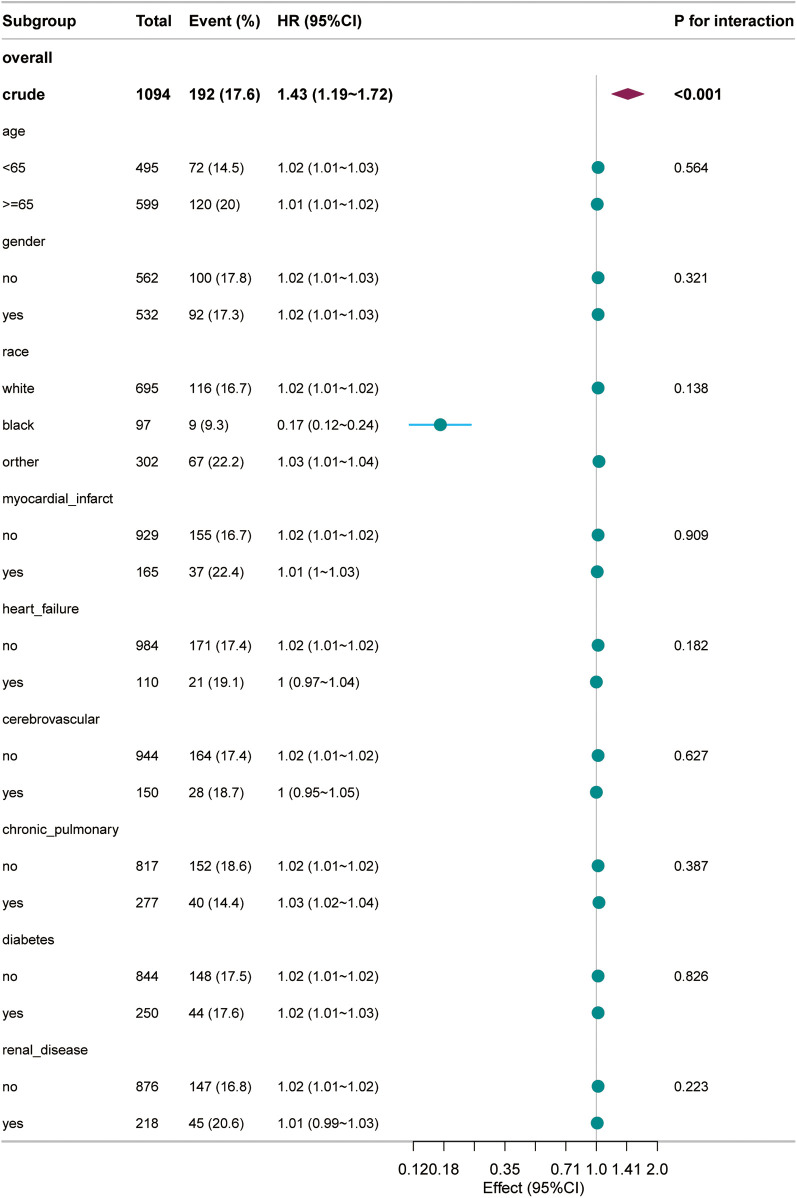
Forest plot for the subgroup analysis of the relationship between 28-day all-cause mortality and blood urea nitrogen.

In the forest plot of this study, the relationship between BUN levels and 28-day all-cause mortality in patients with acute pulmonary edema was found to be generally stable across subgroups. The *P* values for interaction testing were not significant for any subgroup, further confirming the importance of BUN levels as a prognostic marker across different patient characteristics. The forest plots for stratified analysis by age, gender, race, myocardial infarct, heart failure, cerebrovascular disease, chronic pulmonary disease, diabetes, and renal disease showed no significant interaction between BUN levels and each subgroup. Specifically, the *P* values for interaction were 0.564 for age, 0.321 for gender, 0.138 for race, 0.909 for myocardial infarct, 0.182 for heart failure, 0.627 for cerebrovascular disease, 0.387 for chronic pulmonary disease, 0.826 for diabetes, and 0.223 for renal disease. These results demonstrate that BUN is an independent prognostic factor (Figure 4).

## 4 Discussion

Our retrospective cohort study utilizing the MIMIC-IV database provides valuable insights into the association between admission BUN levels and 28-day all-cause mortality in patients with acute pulmonary edema. The findings demonstrate that elevated admission BUN levels are significantly associated with increased mortality risk, highlighting the potential clinical utility of admission BUN monitoring in this patient population.

### 4.1 Mechanisms underlying the association

BUN has traditionally been regarded as a marker of renal dysfunction and metabolic state (13, 14). However, in critically ill patients, BUN's role is more complex as it can also reflect altered cellular metabolism and the body's response to stress (15). In the context of acute pulmonary edema, elevated admission BUN levels may indicate impaired renal function due to reduced renal perfusion and subsequent tissue hypoxia (16). This is supported by our study's observation that higher BUN levels are associated with a greater mortality risk, suggesting that BUN could serve as a surrogate marker for the severity of the underlying condition and the effectiveness of renal function and hemodynamic support.

The dose-response relationship observed in our study, where higher BUN levels correspond to increased mortality risk, underscores the importance of early identification and intervention in patients with elevated BUN. The nonlinear relationship suggested by the restricted cubic spline analysis indicates that the impact of BUN on mortality may vary across different levels, with potentially greater sensitivity at higher thresholds. This finding has implications for clinical decision-making, as it suggests that targeted interventions may be more beneficial in patients with significantly elevated BUN levels.

In the clinical assessment of acute pulmonary edema, plasma natriuretic peptides (e.g., BNP and NT-proBNP) have been widely recognized as important biomarkers for evaluating the severity of heart failure and prognosis (17). These biomarkers primarily reflect the tension of the ventricular walls and cardiac function status. However, BUN levels may complement this information from another perspective. BUN not only reflects renal function but may also be associated with systemic hemodynamics and tissue perfusion. In patients with acute pulmonary edema, elevated BUN levels may indicate impaired renal function or systemic congestion, which is relevant to the pathophysiology of cardiorenal syndrome (18). Therefore, BUN may serve as an indirect indicator of cardiorenal interaction, providing a more comprehensive assessment when used alongside natriuretic peptides. Additionally, compared to serum creatinine, BUN is influenced by multiple factors, including glomerular filtration rate, tubular reabsorption, and protein metabolism. While serum creatinine mainly reflects glomerular filtration function, changes in BUN may more readily reflect changes in renal blood flow, especially when tubular function is impaired (19). Thus, BUN monitoring may complement serum creatinine monitoring, offering clinicians an earlier indication of renal function changes and aiding in the timely adjustment of treatment strategies to improve patient outcomes.

### 4.2 Comparison with previous studies

Previous studies have explored the relationship between BUN levels and outcomes in various critical care settings, including sepsis and heart failure (20–24). However, specific data on acute pulmonary edema are limited. Our study extends these findings by focusing on a cohort of patients with acute pulmonary edema, providing evidence that BUN levels are not only relevant in septic or cardiogenic shock but also in conditions characterized by pulmonary congestion and impaired gas exchange. This aligns with broader observations that BUN can reflect the overall physiological stress and metabolic derangements associated with critical illness. In sepsis research, BUN levels are often closely related to organ dysfunction and inflammatory responses (20); in heart failure studies, changes in BUN levels are associated with the development of cardiorenal syndrome (22); our study further confirms that in patients with acute pulmonary edema, elevated BUN levels may be related to the dual impact of pulmonary circulation impairment and renal dysfunction, providing a new perspective for clinical assessment and intervention.

### 4.3 Clinical implications

The subgroup analysis revealed that the association between BUN levels and mortality was stable across all subgroups. This suggests that BUN levels can be used as a prognostic marker in a diverse patient population, regardless of age, race, gender, or comorbidities. These findings suggest that age-specific or subgroup-specific considerations may not be necessary when using BUN as a prognostic marker, and that a universal management strategy based on BUN monitoring could improve outcomes in patients with acute pulmonary edema.

### 4.4 Limitations and future directions

Despite the strengths of our study, including the large sample size and comprehensive data from the MIMIC-IV database, several limitations should be acknowledged. First, the retrospective nature of the study limits our ability to establish causality. Confounding factors that were not accounted for in the analysis could influence the observed association. Additionally, the study's reliance on a single database may limit generalizability to other populations and settings. There are significant differences in baseline characteristics between BUN quartile groups (e.g., gender, age, heart rate, and renal disease; all with *p* < 0.001). These imbalances may introduce confounding and affect the robustness of the associations reported. Future studies could consider alternative approaches, such as propensity score matching or inverse probability of treatment weighting (IPTW), to adjust for baseline differences and further validate our findings.

## 5 Conclusion

Our study highlights the importance of BUN levels as a predictor of 28-day all-cause mortality in patients with acute pulmonary edema. The findings support the use of BUN as a prognostic marker and emphasize the need for early identification and intervention in patients with elevated BUN levels. Further research is warranted to elucidate the underlying mechanisms and to develop targeted therapeutic strategies based on BUN monitoring.

## Data Availability

The datasets presented in this study can be found in online repositories. The names of the repository/repositories and accession number(s) can be found in the article/Supplementary Material.
